# An Agentic LLM Framework for Autonomous Surgical Continuum Monitoring: ReAct-Driven Tool-Use Agents for Presurgical, Intraoperative, and Postsurgical Cardiopulmonary Care

**DOI:** 10.3390/bioengineering13060686

**Published:** 2026-06-15

**Authors:** Charalampia Pylarinou, Lefteris Gortzis, Vasileios Leivaditis, Elias Liolis, Andreas Antzoulas, Spyros Papadoulas, Konstantinos Nikolakopoulos, Ioannis Panagiotopoulos, Sofoklis Mitsos, Periklis Tomos, Efstratios Koletsis, Francesk Mulita

**Affiliations:** 1 Department of Biomedical Engineering & Biomedical Technology, University of Patras, 26500 Patras, Greece; 2 Department of Medicine, University of Patras, 26504 Patras, Greece; gortzis@med.upatras.gr; 3 Department of Cardiothoracic and Vascular Surgery, Westpfalz Klinikum, 67655 Kaiserslautern, Germany; vnleivaditis@gmail.com; 4 Department of Oncology, University of Patras, 26504 Patras, Greece; lioliselias@yahoo.gr; 5 Department of Surgery, University of Patras, 26504 Patras, Greece; a.antzoulas@hotmail.com; 6 Department of Vascular Surgery, University of Patras, 26504 Patras, Greece; spyros.papadoulas@gmail.com (S.P.); konstantinosn@yahoo.com (K.N.); 7 Department of Cardiac Surgery, Ippokrateio General Hospital of Athens, 11527 Athens, Greece; mdgiapan@yahoo.gr; 8 Department of Thoracic Surgery, Attikon Hospital, National and Kapodistrian University of Athens, 12462 Athens, Greece; sophocmit@yahoo.gr (S.M.); periklistomos@hotmail.com (P.T.); 9 Department of Cardiothoracic Surgery, University of Patras, 26504 Patras, Greece; ekoletsis@upatras.gr

**Keywords:** agentic AI, ReAct framework, reflexion, tool-use LLM agents, surgical continuum, digital twin, clinical decision support, DETER, postsurgical monitoring, HITL, autonomous healthcare AI

## Abstract

Background: Rule-based multi-agent system (MAS) architectures for healthcare coordination rely on hardcoded decision trees that cannot generalise to novel clinical scenarios or self-correct reasoning errors. These limitations are acute in surgical continuum care, where patients traverse presurgical risk stratification, intraoperative monitoring, postsurgical ICU, ward care, and remote rehabilitation over days to weeks—a complexity no fixed-policy agent architecture can address without prohibitive rule engineering. Objective: We present the first agentic large language model (LLM) framework for autonomous end-to-end surgical continuum monitoring, superseding the prior rule-based MAS Digital Twin. Six ReAct-driven tool-use agents replace fixed-policy agents with dynamic reasoning, multi-hop evidence retrieval, and Reflexion self-correction while maintaining mandatory confidence-gated Human-in-the-Loop (HITL) gating at every care-pathway-modifying decision. Methods: The framework is grounded in the ReAct paradigm and Reflexion self-evaluation, embedded within the DETER Digital Twin state engine S(t). Each agent is specified by a ReAct loop signature, a ten-function clinical tool registry, and confidence-gated HITL escalation logic. Inter-agent coordination replaces the rule-based Priority Queue Manager with an LLM-mediated Coordination Supervisor Agent reasoning over competing resource requests. Results: The framework delivers: (i) six formally specified ReAct-loop agents with explicit tool registries and authorisation boundaries; (ii) a confidence-gated HITL architecture that reduces alert fatigue while preserving safety for ambiguous clinical scenarios; (iii) an extended conflict resolution function P(p,t,context) incorporating surgical phase and DETER deterioration trajectory gradient; (iv) Reflexion self-correction with a formal N_max = 2 termination condition and Clinical Factuality Verification Layer; and (v) a multi-phase Digital Twin state engine extending S(t) to the full surgical continuum. Conclusions: The proposed framework represents a fundamental architectural departure from rule-based clinical AI—from hardcoded policies to dynamic reasoning, from static retrieval to multi-hop tool-use chains, and from fixed escalation thresholds to confidence-gated self-evaluation—providing a formally specified, clinically deployable foundation for next-generation autonomous surgical care coordination.

## 1. Introduction

Postsurgical cardiopulmonary care spans one of the most clinically complex and temporally extended patient journeys in medicine: from preoperative risk stratification through operative monitoring, ICU-level postsurgical surveillance, step-down ward care, and finally, remote rehabilitation over weeks to months. Each phase requires coordination across multiple clinical roles—cardiac surgeon, anaesthetist, intensivist, ward nurse, and cardiac rehabilitation specialist—each operating with different data sources, decision horizons, and intervention authorities.

Multi-agent system (MAS) architectures have been proposed for healthcare coordination, including our own prior work extending the DETER framework to a pre-hospital-to-emergency department continuum (Pylarinou and Gortzis, manuscript submitted to Int J Med Inform). These rule-based architectures demonstrate formal logical consistency but share a fundamental limitation: their intelligence is encoded in fixed decision trees and learned PPO policies that cannot generalise beyond scenarios explicitly anticipated during design. When a postsurgical patient presents an unusual combination of arrhythmia, wound infection, and renal impairment three days after surgery—a scenario not in the training distribution—a rule-based agent has no mechanism to reason across the combination. It can only fire the rule closest to the observed pattern.

Definition—Agentic LLM: An agentic LLM is a large language model configured to autonomously pursue goals over multiple steps by interleaving natural language reasoning with the invocation of external computational tools, thereby transcending the single-inference input–output paradigm of standard LLM deployment. This is distinct from an “LLM agent” in the narrow sense (a single LLM performing a bounded task) and from a “multi-agent system” in the classical AI sense (multiple software agents coordinating via message passing under a shared protocol): an agentic LLM framework, as used in this paper, refers specifically to a coordinated architecture of multiple LLM-based reasoning processes, each equipped with a tool registry and self-correction capability, operating within a shared state environment and subject to formal authorisation and HITL safety constraints [ReAct, [1,2]; Reflexion, [3]].

The emergence of agentic large language model (LLM) frameworks provides a principled solution to this generalisation problem. The ReAct paradigm [2]—Reasoning + Acting—enables LLM-based agents to interleave verbal reasoning with tool invocations, generating explicit chain-of-thought justifications for every clinical decision. The Reflexion framework [3] extends this with a self-evaluation loop: agents critique their own reasoning traces and revise their actions before execution, reducing both clinical errors and unnecessary HITL escalations. Together, these paradigms produce agents that are not programmed to handle specific scenarios but are architecturally capable of reasoning about novel ones within constrained tool boundaries.

This paper makes the following specific contributions to the field of clinical AI: [4].

1. **Architectural transformation:** We formally specify the replacement of five rule-based MAS agents with six ReAct-driven tool-use agents, demonstrating how each hardcoded decision function maps to a dynamic reasoning capability.
2. **Surgical continuum adaptation:** We adapt the prior ED-focused architecture to the presurgical-to-post-discharge cardiopulmonary care continuum, redesigning agent roles, tool registries, and HITL gates for the multi-phase surgical journey.
3. **Tool registry specification:** We define a ten-function clinical tool registry with full parameter specifications, enabling agents to invoke DETER deterioration prediction, RAG evidence retrieval, FHIR data access, and resource coordination as runtime tool calls.
4. **Confidence-gated HITL:** We replace fixed escalation thresholds with a confidence-gated HITL architecture where agents self-assess their reasoning quality and escalate to clinicians only when genuinely uncertain—reducing alert fatigue without compromising safety.
5. **Extended conflict resolution:** We extend the prior P(p,t) priority function with surgical-phase and DETER trajectory gradient terms and replace the rule-based Priority Queue Manager with an LLM-mediated Coordination Supervisor Agent.

The remainder of this paper is structured as follows. Section 2 positions the work within the agentic AI and surgical care literature. Section 3 describes the agentic architecture. Section 4 specifies the six agent designs with ReAct loops. Section 5 details the DETER DSS extension to multi-phase surgical knowledge. Section 6 presents the coordination framework, including the extended conflict resolution function. Section 7 discusses formal correctness and safety properties. Section 8 addresses limitations and future directions. Section 9 concludes.

## 2. Background and Related Work

### 2.1. From Rule-Based MAS to Agentic LLM Frameworks

Rule-based multi-agent systems in healthcare have demonstrated value in sub-problem coordination—ICU resource scheduling, appointment management, and clinical pathway optimisation [5]—but their generalisation is fundamentally bounded by the rule set. Classical MAS architectures such as JADE [6] and JASON [7] implement agents as belief–desire–intention (BDI) systems with handcrafted plans. While formally verifiable, these architectures cannot adapt to clinical scenarios outside their plan library.

Agentic LLM frameworks represent a paradigm shift from plan execution to plan generation. The ReAct framework [2] demonstrated that LLMs interleaving verbal reasoning with tool use outperform both pure chain-of-thought reasoning and pure tool invocation on knowledge-intensive tasks because the verbal reasoning step allows the model to assess partial results and redirect its tool strategy. Reflexion [3] adds a second level of abstraction: after generating an action plan, the agent generates a verbal self-critique of its own reasoning, revising the plan before execution. Applied to clinical settings, this creates an agent that asks not only “what should I do?” but “am I confident enough in this reasoning to act without human confirmation?” This is precisely the question that clinical AI safety requires, which no rule-based system can answer [8].

### 2.2. Surgical Continuum Coordination: The Unsolved Problem

The surgical care continuum from consent to rehabilitation has received substantially less attention than acute ED and ICU coordination in the AI literature. Existing approaches either address isolated phases—preoperative risk scoring [9], intraoperative anaesthesia decision support [10], and ICU early warning systems [1]—or propose generic care pathway engines without phase-specific agent logic [11]. No prior work has proposed an autonomous multi-agent architecture that coordinates reasoning and action across all surgical phases with shared state, dynamic tool use, and formal safety constraints.

The DETER framework [1] provides the continuous deterioration prediction engine that constitutes the core monitoring capability of the present work. Pylarinou et al. [12] established a constraint optimisation formalism for pre-hospital scheduling that informs the extended conflict resolution function. The present paper extends both contributions into an agentic LLM architecture that replaces the rule-based agent logic of the prior MAS framework [1] while retaining the formal Digital Twin state engine and HITL safety architecture [13,14].

### 2.3. LLM Tool Use in Clinical Settings

LLM-based clinical tool use is an active research frontier. Med-PaLM 2 [15] demonstrated expert-level medical question answering but without structured tool invocation. GPT-4 function calling and the emerging OpenAI Assistants API provide the technical mechanism for structured tool registration and runtime invocation. BioMistral-7B [16]—used in our prior work—provides a GDPR-compliant on-premise LLM suitable for production clinical deployment. AutoGen [17] and CrewAI provide multi-agent orchestration frameworks compatible with ReAct-style agent loops. The clinical application of these frameworks to surgical continuum coordination, with formally specified tool registries and confidence-gated HITL, is the specific contribution of this paper [18,19,20,21,22,23,24,25].

Table 1 positions the proposed framework against six representative prior clinical AI coordination systems across seven architectural dimensions. The autonomy levels are L1 = rule-bound, L2 = closed-loop parametric, L3 = single-inference LLM, L4 = dynamic multi-agent LLM, and L5 = agentic LLM with self-correction. CFVL = Clinical Factuality Verification Layer.

The proposed framework is the only system combining formal tool-use safety, mandatory confidence-gated HITL, Reflexion self-correction with CFVL, and full presurgical-to-rehabilitation temporal coverage.

### 2.4. Reflexion and Self-Evaluation in Healthcare AI

The Reflexion framework [3] extends ReAct with verbal reinforcement: agents generate a natural language self-critique of each reasoning trace, storing the critique in an episodic memory buffer that informs future reasoning on similar problems. In clinical AI, this self-evaluation property is particularly valuable because it provides a mechanism for agents to express uncertainty and request human input rather than proceeding with low-confidence actions. Unlike calibrated uncertainty in discriminative classifiers—which requires distributional assumptions—Reflexion-based self-evaluation uses the LLM’s own reasoning capacity to assess confidence, a property that generalises to novel scenario types by construction.

Hallucination Mitigation—RAG Provenance Cross-Referencing and Clinical Factuality Verification Layer (CFVL): Reflexion self-evaluation reduces reasoning errors but cannot detect factual errors that are internally coherent. Two additional safeguards are therefore incorporated between the Reason and Act steps. First, in the RAG provenance cross-referencing step, every clinical factual claim in an agent’s reasoning trace that references a guideline recommendation, drug dosage, complication incidence figure, or risk score threshold is tagged by RAG_retrieve() with a provenance identifier linking it to the source document and section. A lightweight factual grounding checker identifies any factual claim lacking a provenance tag (generated from parametric LLM memory rather than retrieved evidence) and flags it for explicit HITL review. Second, the CFVL checks three factual consistency conditions before permitting the Act step: (i) numerical values (drug dosages, risk thresholds, vital sign reference ranges) are validated against a curated clinical reference table in the DETER DSS; (ii) cited guideline recommendations are cross-referenced against the RAG knowledge base; and (iii) proposed clinical actions are checked against the BNF/RxNorm contraindication index for the patient’s current medications. If the CFVL detects a discrepancy, the reasoning trace is flagged as “unverified factual content”, and HITL_escalate() is mandatory regardless of the agent’s expressed confidence.

## 3. Agentic Digital Twin Architecture

### 3.1. Architectural Overview

**Figure 1:**
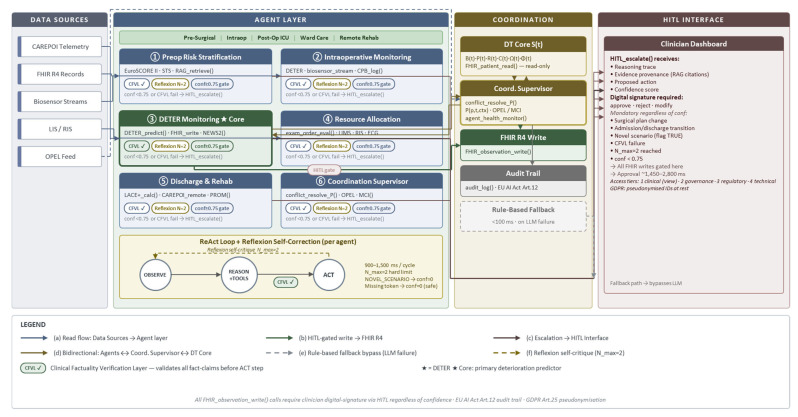
System-level data flow and agent orchestration. Four lanes: (1) data sources—CAREPOI telemetry, FHIR R4, biosensor streams, LIMS/RIS, OPEL; (2) agent layer—six agents with surgical-phase activation bands; (3) coordination layer—Coordination Supervisor, DT Core S(t), and conflict_resolve_P(); (4) HITL interface. Arrow types: (a) read flows; (b) HITL-gated writes to FHIR and audit trail; (c) escalation arrows to HITL; (d) dashed rule-based fallback bypass; (e) Reflexion self-critique loop (N_max = 2). CFVL gate shown inline between REASON and ACT.

The proposed architecture retains the seven-layer Healthcare Digital Twin structure of the prior framework [1] but replaces the rule-based agent logic at Layers 5–7 with agentic LLM coordination. The Digital Twin Core state engine S(t), the data ingestion and processing layer, and the HITL interface layer are preserved (Figure 1). The fundamental change is at the agent computation layer: each agent is no longer a state machine executing predefined plans but an LLM-based reasoning process that observes its environment, reasons about the appropriate action, invokes tools from its registry, evaluates its own reasoning, and acts within authorised boundaries [18,26].

The formal state space is extended from the prior formulation to accommodate the surgical continuum:

S(t) = {B(t), P(t), R(t), C(t), Ω(t), Φ(t)}

where the new term Φ(t) = {phase_i(t), surgical_procedure_i, anaesthesia_record_i, CPB_events_i, wound_status_i} captures the surgical-phase context vector for each active patient. This extension enables surgical-phase-aware reasoning by all agents without architectural modification: Φ(t) is read-accessible by all agents through the FHIR_patient_read() tool call.

### 3.2. The ReAct Loop as Agent Computation Model

Each agent in the framework is implemented as a ReAct loop [2] operating on the Digital Twin state and the agent’s tool registry. The loop has three steps executed sequentially:

1. **Observe:** The agent reads its relevant state slice from S(t) via authorised FHIR tool calls. For the DETER Monitoring Agent, this includes the current 144-step physiological window, recent EMR deltas, and active DETER risk scores. For the Coordination Supervisor, this includes all agent heartbeat states and the current resource competition queue.
2. **Reason:** The agent generates a verbal chain-of-thought reasoning trace over the observed state, drawing on its domain-specific system prompt, the retrieved evidence from RAG_retrieve(), and the outputs of any computational tools invoked during reasoning. The reasoning trace is explicitly recorded in the audit log for every cycle.
3. **Act:** The agent selects and invokes the appropriate action from its tool registry. Before any tool execution that modifies the care pathway—FHIR_observation_write(), bed_assign(), examination_order_evaluate(), and alert_escalate()—the agent invokes HITL_escalate() if its self-assessed confidence is below the configured threshold (default: 0.75) or if the action type is flagged as requiring mandatory human confirmation.

Following the Act step, the Reflexion self-evaluation is invoked. The agent generates a verbal critique of its own reasoning trace: “Did I consider all relevant observations? Was the tool I invoked appropriate? Is my confidence well-calibrated for this scenario?” The critique is stored in the agent’s episodic memory buffer (maintained across the surgical episode) and referenced in future cycles. If the critique identifies a reasoning error, the agent re-executes the Reason step with the critique as additional context before proceeding to Act.

Reflexion Loop Termination Conditions: The Reflexion loop terminates on any of three conditions: (1) convergence—revised reasoning trace yields self-assessed confidence ≥ θ_convergence = 0.75 with no identified factual errors; (2) hard iteration limit—N_max = 2 revision cycles unconditionally, regardless of convergence; and (3) CFVL failure—an unresolvable factual discrepancy that additional reasoning cannot correct. On reaching the hard limit or CFVL failure without convergence, HITL_escalate() is invoked mandatorily and non-convergence is recorded in the audit trail. For the Intraoperative Monitoring Agent, N_max = 2 is applied consistently with all other agents. The value N_max = 2 is justified by Reflexion [3]: the majority of self-correction gains accrue in the first revision cycle; a second adds marginal benefit at 800–1500 ms additional latency, after which the HITL path is more efficient.

### 3.3. Confidence-Gated HITL Architecture

A critical departure from the prior framework’s fixed escalation thresholds is the confidence-gated HITL mechanism. In the prior architecture, HITL was triggered by threshold breaches in the priority function P(p,t) or by explicit clinical alert levels (NEWS2 ≥ 5). In the agentic framework, every agent maintains a running confidence estimate for its current reasoning cycle, updated at each Observe–Reason–Act iteration. Mandatory HITL escalation is triggered by any of four conditions:

- **Confidence gate:** agent self-assessed reasoning confidence < 0.75 at the Act step, as expressed in the Reflexion self-critique.
- **Novel scenario detection:** agent identifies in its reasoning trace that the current clinical presentation is outside its training distribution or its episodic memory of prior cases.
- **Mandatory action types:** any tool execution in a designated mandatory HITL category regardless of confidence: surgical plan modifications, admission-to-discharge transitions, and anaesthesia protocol recommendations.
- **Priority tie:** inter-agent resource conflict where |P(p,t) − P(p’,t)| < ε_tie = 0.01 after LLM-mediated reasoning by the Coordination Supervisor.

This architecture reduces alert fatigue relative to the prior framework by eliminating HITL escalation for high-confidence routine actions—a postsurgical patient with stable trending vitals at 24 h does not require human confirmation to log a routine DETER observation. However, it strengthens HITL for genuinely ambiguous scenarios by anchoring escalation to the agent’s expressed reasoning quality rather than to fixed threshold parameters.

#### Confidence Score Elicitation Mechanism

Confidence scores are elicited via a mandatory instruction appended to each agent’s system prompt: “Output your self-assessed confidence as a decimal in [0.0, 1.0] using the format CONFIDENCE: X.XX. Output NOVEL_SCENARIO: TRUE if the presentation is outside your training distribution, otherwise FALSE.” The values are extracted by a deterministic regex parser, not a second LLM call. Safe-failure defaults: missing CONFIDENCE token → confidence = 0.0 (mandatory HITL); NOVEL_SCENARIO: TRUE → confidence floored to 0.0. Raw self-assessment is not a calibrated probability; Platt scaling and isotonic regression on the 94-patient DETER cohort (Section 7.3) provide empirical calibration.

Table 2 presents the quantitative fallback trigger thresholds governing the transition from LLM-mediated agentic reasoning to rule-based fallback execution for each agent and clinical context.

## 4. Agentic Agent Design

Table 3 presents the fundamental architectural contrast between the prior rule-based MAS framework and the proposed agentic LLM framework across eight dimensions.

Table 4 specifies each of the six agentic agents by surgical phase, ReAct loop signature, tool registry, and authorisation/HITL gate.

### 4.1. Preoperative Risk Stratification Agent

The Preoperative Risk Stratification Agent is the first agent in the surgical continuum, activated on surgical consent. Its primary function is to synthesise a comprehensive perioperative risk profile from the patient’s EHR, structured comorbidity data, and current cardiopulmonary status, grounded in validated risk stratification instruments.

**ReAct loop:** The agent observes the patient’s FHIR bundle via FHIR_patient_read(), invoking EuroSCORE_calc() and the STS risk calculator API to generate quantitative risk estimates for 30-day mortality, stroke, renal failure, and prolonged ventilation. RAG_retrieve() is invoked with a phase_filter parameter set to “presurgical” to retrieve the most relevant procedure-specific outcome profiles from the 97-procedure cardiopulmonary surgery evidence base. The reasoning trace synthesises quantitative scores with retrieved evidence to generate a structured risk report with confidence intervals. The report is presented to the surgical team via HITL_escalate()—categorised as a mandatory action type—before being written to the FHIR surgical plan record. If the agent’s Reflexion self-critique identifies a missing data element (e.g., no recent echocardiogram), it generates a structured data request before producing the risk report.

**Authorisation boundary:** The agent may read all FHIR patient resources and generate risk reports. It may not modify the surgical plan or schedule directly. All surgical plan endorsements or high-risk flags require clinician digital-signature confirmation via HITL_escalate().

### 4.2. Intraoperative Monitoring Agent

The Intraoperative Monitoring Agent operates during the operative phase, processing real-time haemodynamic data, anaesthesia depth monitoring signals, and cardiopulmonary bypass (CPB) events. It replaces and substantially extends the capabilities of the prior framework’s Monitoring Agent, which was designed for continuous vital sign streaming but not for the high-frequency, multimodal intraoperative environment.

**ReAct loop:** The agent observes biosensor streams via biosensor_stream_read() and anaesthesia monitor outputs via anaesthesia_monitor_api() at configurable intervals (default: 60 s observation window). DETER_predict() is invoked on the current physiological window to generate intraoperative deterioration probability estimates. The reasoning trace evaluates trajectory against operative stage: haemodynamic instability during CPB weaning is interpreted differently from equivalent instability during skin closure. CPB events—cross-clamp time, reperfusion, de-airing—are logged via CPB_event_log() and contribute to the Φ(t) surgical context vector. Alerts are published via alert_publish() to the Coordination Supervisor and Resource Allocation Agent, with confidence-gated HITL escalation for any anaesthesia protocol suggestion.

### 4.3. DETER Monitoring Agent (Core)

The DETER Monitoring Agent is the continuous thread connecting all postsurgical phases, directly implementing the DETER deterioration prediction algorithm [1] within the agentic framework. It operates continuously from ICU admission through ward discharge, generating personalised 6 h/24 h/7-day risk scores at every 5–10 min telemetry cycle.

**ReAct loop:** The agent observes the current 144-step physiological window from CAREPOI^®^ telemetry alongside EMR delta records via FHIR_patient_read(). DETER_predict() generates a risk score vector with confidence intervals and feature importance map. RAG_retrieve() is invoked when the risk score crosses a configurable threshold (default: >0.6 at 6 h horizon) to retrieve relevant cardiopulmonary surgery evidence supporting the predicted deterioration pathway. The reasoning trace synthesises the DETER prediction with retrieved evidence and the patient’s surgical context Φ(t) to generate a structured clinical recommendation. FHIR_observation_write() records the observation and recommendation to the patient record. NEWS2_calc() provides a comparative EWS overlay for clinician reference. audit_log() records the complete reasoning chain for every cycle.

**Reflexion self-correction:** If the agent’s self-critique identifies that the current prediction conflicts with the prior 24 h trajectory in a physiologically implausible way—e.g., a sudden AUC jump from 0.3 to 0.9 without corresponding vital sign changes—it flags a potential artefact, invokes FHIR_patient_read() for recent medication administration records, and revises its reasoning before alerting. This self-correction mechanism substantially reduces false positive deterioration alerts from sensor artefacts or expected postoperative physiological responses.

### 4.4. Resource Allocation Agent

The Resource Allocation Agent is retained from the prior framework [1], but its rule-based examination rationalisation logic is replaced by LLM-mediated evaluation of each examination order against a dynamically retrieved evidence base. This enables phase-appropriate examination protocols that adapt to the patient’s current surgical phase rather than applying fixed ESI-stratified rules designed for the acute ED context.

**ReAct loop:** The agent observes the current DT Core resource state via DT_state_read() and incoming examination orders from all clinical agents. examination_order_evaluate() is invoked for each order, querying the evidence base for the examination’s appropriateness given the patient’s current surgical phase, DETER risk score, and resource availability. The reasoning trace generates a structured justification for each approve/flag/redirect decision. For patients in the postsurgical ICU phase with elevated DETER scores, the agent applies a higher-sensitivity protocol; for step-down ward patients with stable DETER trajectories, a more conservative protocol reduces unnecessary testing. ECG_coordinate() is invoked for patients meeting HEART protocol criteria, ensuring cardiac risk screening is not omitted even when examination panels are rationalised.

Illustrative Example—Monitoring Agent vs. Resource Allocation Agent Conflict: The DETER Monitoring Agent requests an urgent echocardiogram for a patient with DETER 6 h risk score = 0.73 (rising, ∇DETER = 0.18 over two cycles). The Resource Allocation Agent flags the order as low priority based on current echo slot availability. conflict_resolve_P() is invoked: the patient’s P(p,t,context) score receives w_5_·f_5_(∇DETER = 0.18) = 0.10 × 0.18 = 0.018; the resulting total priority advantage over the competing resource request exceeds the auto-resolution threshold ε_auto = 0.05. Stage 1 resolution: the echo is expedited automatically. Total conflict-to-resolution time: approximately 1800 ms. This example is elaborated as Scenario 4 in Section 8.4.

### 4.5. Discharge and Rehabilitation Agent

The Discharge and Rehabilitation Agent operates at the post-discharge boundary, managing the critical transition from inpatient monitoring to remote CAREPOI^®^ telemetry. It replaces the prior framework’s Discharge/Admission Agent with a substantially expanded role covering rehabilitation trajectory assessment and patient-reported outcome integration.

**ReAct loop:** The agent observes the LACE+ readiness score via LACE_plus_calc(), current DETER 7-day risk trajectory, and patient-reported symptoms via PROM_schedule() questionnaire data. RAG_retrieve() is invoked for discharge timing evidence specific to the patient’s surgical procedure and risk profile. The reasoning trace synthesises clinical and patient-reported data to generate a structured discharge plan with remote monitoring intensity recommendation. CAREPOI_remote_init() activates the remote monitoring protocol at the appropriate intensity level. EHR_writeback() closes the inpatient record and initiates the remote monitoring episode. Mandatory HITL confirmation is required before the admission-to-discharge transition regardless of confidence level.

### 4.6. Coordination Supervisor Agent

The Coordination Supervisor Agent replaces the prior framework’s rule-based Tier 2 Priority Queue Manager. It is the only agent whose primary function is inter-agent coordination rather than direct patient care, and it is the agent most fundamentally transformed by the shift to agentic LLM reasoning.

**ReAct loop:** The agent observes the health status of all active agents via agent_health_monitor(), the current DT Core resource competition queue, and the OPEL surge level. conflict_resolve_P() is invoked to compute the extended priority function P(p,t,context) for each competing resource request. The reasoning trace generates a chain-of-thought justification for each prioritisation decision, explicitly citing the component scores and the contextual factors that influenced the weighting. HITL_escalate() is invoked when the LLM-mediated reasoning fails to resolve a conflict—i.e., when the agent’s Reflexion critique identifies that the available information is insufficient for a confident decision. MCI_protocol_trigger() is invoked when OPEL exceeds the configured surge threshold, dynamically instantiating an MCI Coordinator sub-agent and suspending elective surgical pathways.

## 5. Clinical Tool Registry

The tool registry is the interface between agent reasoning and clinical action. Each tool is a formally specified function with typed input parameters, defined return schema, authorisation requirements, and audit logging. Table 5 presents the full ten-function registry.

### 5.1. Tool Security and Authorisation Architecture

Tool invocation is governed by a three-layer authorisation model. At Layer 1, each tool is assigned a scope (read|write|escalate) and a minimum authorisation level (autonomous|HITL-gated|mandatory-human). At Layer 2, each agent has a statically configured tool registry that specifies which tools it may invoke and under what conditions. An agent cannot invoke a tool outside its registry regardless of reasoning content. At Layer 3, the DETER DSS clinical safety filter validates every tool call against the invoking agent’s authorisation boundary before execution, logging any attempt to exceed authorisation scope to the immutable audit trail.

This three-layer model ensures that even if an LLM agent generates a reasoning trace that appears to justify a tool call outside its authorisation boundary—a risk inherent in generative reasoning—the call is rejected at Layer 3 with a structured error message returned to the agent’s reasoning loop. The agent can then revise its reasoning within its authorisation constraints or invoke HITL_escalate() to request expanded authority for the specific situation.

#### Adversarial Threat Model

Four threat categories are addressed by the three-layer authorisation model. (1) Prompt injection via FHIR free-text fields: mitigated by Layer 3 input sanitisation, adversarial-robustness system-prompt engineering, and Layer 2 whitelist rejection of any out-of-registry tool call regardless of source. (2) Adversarial tool outputs: CFVL validates all numerical outputs against physiologically plausible ranges before they enter the reasoning trace; out-of-range values trigger HITL with a data-integrity flag. (3) Privilege escalation: out-of-registry call attempts are rejected at Layers 2 and 3 and written to the immutable audit trail. (4) Lateral agent manipulation: all FHIR writes require clinician digital-signature confirmation via HITL_escalate(), preventing any agent from writing manipulated state to the shared DT Core.

Inter-Agent Directive Conflict Resolution—Four-Stage Logic: When specialised agents issue conflicting directives (e.g., the DETER Monitoring Agent orders an urgent investigation that the Resource Allocation Agent flags as resource-inappropriate), the Coordination Supervisor resolves the conflict in four stages:

Stage 1—Automatic resolution via P(p,t,context): If the priority score difference between the two positions exceeds ε_auto = 0.05, the higher-priority directive executes. A rapidly rising DETER gradient (f_5_ term) typically overrides a resource flag.

Stage 2—LLM-mediated reasoning: If the priority difference < ε_auto = 0.05, the Coordination Supervisor generates a chain-of-thought reasoning trace weighing both positions against RAG-retrieved evidence, producing a structured resolution proposal with explicit justification.

Stage 3—Automatic HITL for unresolved conflicts: If the Coordination Supervisor’s Reflexion self-critique yields confidence < 0.75 after N_max = 2 reasoning iterations, HITL_escalate() is automatically triggered. The clinician receives both agents’ positions, priority function scores, and the Coordinator’s reasoning trace. The clinician’s decision is binding and written to the immutable audit trail.

Stage 4—Mandatory HITL categories: Two inter-agent conflict categories always trigger mandatory HITL regardless of priority score or Coordinator confidence: (i) conflicts involving admission-to-discharge transitions; (ii) conflicts involving anaesthesia or surgical protocol modifications.

### 5.2. DETER DSS Extension to Multi-Phase Surgical Knowledge

The DETER DSS RAG pipeline [1] is extended from a five-source triage-focused knowledge base to a seven-source multi-phase surgical knowledge graph:

- **Cardiopulmonary surgery literature (PubMed):** 97 procedure-specific outcome profiles with temporal evidence weighting [31].
- **Clinical practice guidelines:** ESC, AHA/ACC, STS guidelines for cardiac, thoracic, and major vascular surgery; NICE perioperative guidelines.
- **FHIR patient state records:** real-time S(t) slice from the DT Core, including Φ(t) surgical context vector.
- **SNOMED-CT + ICD-11:** coded clinical concept matching for structured query disambiguation.
- **BNF/RxNorm drug-interaction safety index:** postsurgical polypharmacy interaction screening.
- **Anonymised institutional outcome data:** procedure-matched prior cases from the University of Patras Cardiothoracic Clinic, indexed by EuroSCORE II risk decile and procedure type.
- **DETER prediction history:** the current patient’s own DETER risk score trajectory, enabling retrieval of similar physiological trajectories from anonymised prior cases for comparative context.

The RAG pipeline implements multi-hop retrieval for complex clinical questions. When the DETER Monitoring Agent’s reasoning identifies a potential complication (e.g., “elevated troponin trend + new atrial fibrillation + low cardiac output”), a multi-hop chain is executed: (1) retrieve complication profiles matching the symptom combination; (2) retrieve the procedure-specific incidence data for this combination; (3) retrieve guideline recommendations for the identified complication pattern; and (4) retrieve similar cases from institutional outcome data. The assembled multi-hop context is significantly more clinically informative than single-query retrieval, reducing the risk of LLM generation being disconnected from the patient’s specific surgical context.

## 6. Agentic Coordination Framework

### 6.1. Three-Tier Structure Retained and Extended

The three-tier hierarchical coordination structure of the prior framework [1] is retained but substantively modified at each tier. Tier 1 individual agents now operate as ReAct loops with Reflexion self-correction rather than as PPO-trained or rule-based state machines. Tier 2 inter-agent coordination is managed by the Coordination Supervisor Agent operating as an LLM reasoner over the priority function and resource competition queue rather than a deterministic Priority Queue Manager. Tier 3 system-wide meta-optimisation is extended to include monitoring of agent reasoning quality—tracking the frequency of HITL escalations, the rate of Reflexion self-corrections, and the confidence distribution across agents over time—providing a meta-level signal for identifying agents that are underperforming or encountering systematic reasoning failures.

### 6.2. Extended Conflict Resolution Function P(p,t,context)

The prior conflict resolution function P(p,t) = w_1_·f_1_(ESI) + w_2_·f_2_(WT) + w_3_·f_3_(CR) is extended with two surgical-continuum-specific terms:


**P(p,t,context) = w_1_·f_1_(acuity_p) + w_2_·f_2_(WT_p(t)) + w_3_·f_3_(CR_p(t)) + w_4_·f_4_(phase_p(t)) + w_5_·f_5_(**
**∇**
**DETER_p(t))**

The three inherited terms are redefined for the surgical continuum context:

f_1_(acuity_p): patient clinical acuity score, normalised to [0, 1]. The value is computed as a weighted composite: acuity_p = 0.5·DETER_6h(t) + 0.3·NEWS2_p(t)/20 + 0.2·EuroSCORE_p/100, where DETER_6h(t) is the current 6 h deterioration risk score (range 0–1), NEWS2_p(t) is the current National Early Warning Score 2 (range 0–20), and EuroSCORE_p is the patient’s preoperative EuroSCORE II (range 0–100). This replaces the Emergency Severity Index (ESI) used in the prior ED triage formulation, reflecting the multi-dimensional acuity assessment available across the surgical continuum.

f_2_(WT_p(t)): time-urgency score, normalised to [0, 1]. In the surgical continuum context, WT_p(t) represents the elapsed time since the most recent clinical assessment or care event for patient p, normalised against a phase-specific maximum expected assessment interval: WT_p(t) = min(1.0, time_since_last_assessment_p(t)/T_max_phase), where T_max_phase is 480 min (ICU), 360 min (ward), or 60 min (intraoperative). This replaces the ED queue waiting time, which is not applicable in the inpatient or remote monitoring context.

f_3_(CR_p(t)): postoperative complication risk modifier, normalised to [0, 1]. CR_p(t) captures the patient’s cumulative complication risk based on documented postsurgical events: CR_p(t) = min(1.0, Σ_i_ severity_weight(event_i)), where each logged complication event (wound infection, arrhythmia, AKI, respiratory failure, re-intubation) contributes a severity weight in the range [0.1, 0.4] derived from the STS complication severity classification. Patients with active complications receive elevated priority scores independent of their current vital sign trajectory.

The new terms are:

**f_4_(phase_p(t)):** surgical phase urgency modifier. Values: 0.0 (preoperative stable), 0.3 (postsurgical > 72 h stable), 0.6 (postsurgical 24–72 h), and 0.9 (immediate postoperative <24 h or intraoperative). This term ensures that immediate postoperative patients receive higher resource priority than equivalent acuity patients at later phases, reflecting the well-established higher complication rate in the first 24 postoperative hours.

**f_5_(∇DETER_p(t)):** DETER deterioration trajectory gradient. This term captures the average rate of change of the DETER 6 h risk score over the past two prediction intervals (spanning three prediction cycle timestamps): ∇DETER_p(t) = (DETER_6h(t) − DETER_6h(t − 2Δ))/(2Δ), normalised to [0, 1]. A patient with a rapidly rising DETER score is prioritised over a patient with an equivalently elevated but stable score because the rising trajectory indicates active deterioration rather than stable elevated risk. The normalisation bounds are: gradient values above +0.15/Δ (rapid deterioration) are clamped to 1.0; values below −0.15/Δ (rapid improvement) are clamped to 0.0. Negative gradient values (improving trajectory) are floored at 0.0 so as not to penalise improving patients.

Default weights: w = (0.40, 0.15, 0.20, 0.15, 0.10), reflecting the primacy of clinical acuity in the surgical context, with reduced waiting-time weight compared to the ED formulation (where queue time is a primary clinical concern) and increased surgical phase and trajectory terms.

Table 6 presents the full comparison between prior and extended conflict resolution components.

### 6.3. Surgical Phase as Coordination Anchor

In the prior framework, triage (ESI assignment) served as the coordination anchor: all agents oriented their priorities around the ESI level assigned by the ED Triage Agent. In the surgical continuum, the coordination anchor is replaced by the patient’s surgical phase Φ(t) and their current DETER trajectory. The surgical phase determines which agents are active, what tool invocations are appropriate, and how resource conflicts are weighted. This phase-based coordination model enables the agentic framework to naturally handle the surgical continuum without requiring explicit inter-phase handoff protocols: agents observe Φ(t) from the DT Core and adapt their reasoning accordingly.

### 6.4. Scalability: Dynamic Agent Instantiation

A structural advantage of the agentic LLM architecture over the prior rule-based MAS is the ability to instantiate specialised sub-agents at runtime without modifying the base architecture. The Coordination Supervisor Agent can instantiate a Paediatric Cardiac Surgery Sub-Agent, a Transplant Rejection Monitoring Sub-Agent, or an MCI Coordinator Sub-Agent dynamically when the surgical context demands it by providing the base LLM with a specialised system prompt and a restricted tool registry for the sub-agent role. These sub-agents operate within the same HITL and authorisation architecture as the primary agents, requiring no architectural modification or retraining.

## 7. Safety Properties and Formal Specifications

### 7.1. Safety Invariants

The agentic framework maintains four safety invariants that are enforced architecturally rather than by LLM reasoning:

1. **No care-pathway modification without HITL confirmation:** Any tool execution in the {FHIR_observation_write, bed_assign, EHR_writeback, CAREPOI_remote_init, MCI_protocol_trigger} set requires a prior HITL_escalate() call with a clinician digital signature. This invariant is enforced at Layer 3 of the tool authorisation architecture, not by the agent’s reasoning logic.
2. **No tool invocation outside agent registry:** The Layer 2 tool registry whitelist is immutable at runtime. Agents cannot invoke tools not in their registry, and the DETER DSS clinical safety filter rejects out-of-registry calls before execution.
3. **Immutable audit trail:** Every Observe–Reason–Act cycle, every tool call (attempted and executed), every Reflexion self-critique, and every HITL outcome is written to the DETER DSS audit trail via audit_log(). The audit trail is append-only and cryptographically signed (EU AI Act Article 12 compliance).
4. **Graceful degradation:** If any agent fails (timeout, exception, or LLM inference failure), the Coordination Supervisor activates a backup rule-based fallback agent for that role. The fallback agent implements the equivalent of the prior framework’s rule-based logic, ensuring patient safety is maintained even during LLM infrastructure failures. The fallback activation is logged and triggers a HITL alert.

### 7.2. Formal State-Space Preservation

The extended Digital Twin Core state space S(t) = {B(t), P(t), R(t), C(t), Ω(t), Φ(t)} with the hybrid event/time-driven update operator S(t + Δt) = 𝒯(S(t), A(t), E(t)) is formally preserved from the prior framework. The agentic architecture does not modify the state transition function; it changes the computation of A(t)—the vector of agent actions—from rule-based policy evaluation to ReAct-loop output. State consistency guarantees therefore depend on the tool authorisation architecture (which gates writes to S(t)) rather than on the LLM reasoning process.

### 7.3. Confidence Threshold Calibration Protocol

The HITL confidence threshold (default: 0.75) is a pre-calibration conservative estimate. Empirical calibration using the 94-patient DETER validation cohort [4] is a prerequisite for clinical deployment. The calibration protocol proceeds as follows:

Dataset: Each DETER_predict() inference from the 94-patient cohort is paired with the agent’s self-assessed confidence score (expressed numerically in the Reflexion self-critique) and the ground-truth clinical outcome (deterioration or stable) at the 6 h horizon.

Method 1—Platt scaling: A logistic regression is fitted with self-assessed confidence as the input and the binary deterioration outcome as the label. The sigmoid transformation maps raw self-assessed confidence to a calibrated probability estimate, correcting for systematic LLM overconfidence or underconfidence.

Method 2—Isotonic regression: A non-parametric monotone function is fitted between self-assessed confidence and observed accuracy without assuming a functional form. Isotonic regression is preferred when the raw confidence–accuracy relationship is non-monotone over parts of the range, as may occur for novel scenario types.

Threshold selection: Following calibration, the optimal HITL threshold minimises a clinical loss function L = α·FN + β·FP, where FN (failing to escalate when clinician input was needed) is weighted more heavily than FP (unnecessary escalation). Initial weighting α/β = 3 reflects the asymmetric cost of missed deterioration versus alert fatigue; sensitivity analysis across α/β ∈ {2, 3, 5} will determine robustness.

### 7.4. EU AI Act High-Risk Compliance Pathway

The DETER Agentic Framework falls under the EU AI Act Annex III high-risk classification (Article 6, paragraph 2: AI systems intended to assist clinical decisions that may affect individual health or safety). The following architectural properties directly address the technical documentation requirements:

- **Transparency and explainability (Article 13):** Every agent reasoning trace is recorded verbatim in the audit trail; every tool call is logged with input parameters and output; HITL confirmations are digitally signed and timestamped [8].
- **Human oversight (Article 14):** HITL_escalate() is a mandatory architectural gate for all care-pathway modifications; clinicians have one-click override capability at every escalation point; the confidence-gated HITL ensures human oversight is proportionate to clinical uncertainty.
- **Accuracy, robustness, and cybersecurity (Article 15):** Reflexion self-correction improves reasoning robustness; the rule-based fallback agent ensures continuity of function during LLM failures; the tool authorisation architecture prevents prompt-injection attacks from influencing tool execution.
- **Data governance (Article 10):** BioMistral-7B or Meditron-70B on-premise deployment ensures no patient data egress to third-party LLM providers; all FHIR data access is governed by hospital IG policies and GDPR Article 25 privacy-by-design.

### 7.5. Reasoning Trace Access Control and Governance

Stored reasoning traces require explicit governance. Access is stratified into four tiers: Tier 1 (clinical staff) sees only the structured HITL dashboard output; Tier 2 (clinical governance) may access full traces for quality assurance under hospital IG policy; Tier 3 (regulatory audit) accesses immutable traces under EU AI Act Article 12 on formal request; and Tier 4 (technical teams) accesses pseudonymised traces for safety analysis under data-sharing agreements. Patient identifiers are pseudonymised at rest under GDPR Article 9(1). Reasoning traces constitute AI system records, not clinical documentation; their medico-legal discoverability in litigation varies by jurisdiction and should be confirmed with hospital legal counsel prior to deployment.

## 8. Limitations and Future Directions

This paper presents a proposed agentic architecture; it does not report a prospective clinical implementation. Four categories of limitation are acknowledged:

### 8.1. LLM Reasoning Reliability

LLM-based agents are susceptible to hallucination—generating confident-sounding but factually incorrect clinical reasoning. The tool authorisation architecture and Reflexion self-correction reduce but do not eliminate this risk. BioMistral-7B and Meditron-70B achieve near-GPT-4 performance on medical benchmarks (MedQA, MedMCQA) [32] but have not been validated on postsurgical cardiopulmonary clinical reasoning tasks or on Greek-language clinical notes. Systematic hallucination testing using adversarial clinical scenarios is required before pilot deployment.

Explicit Caveat on Foundation Model Benchmarks (NEW): The performance of BioMistral-7B and Meditron-70B on general medical benchmarks (MedQA, MedMCQA) does not imply validated readiness for postsurgical cardiopulmonary clinical reasoning. These benchmarks evaluate knowledge recall and multiple-choice reasoning across broad medical domains; they do not assess temporal reasoning over physiological trajectories, integration of intraoperative event logs with postsurgical deterioration patterns, or the specific clinical language and protocols of cardiopulmonary surgery. Domain-specific fine-tuning on de-identified cardiopulmonary surgery cases and rigorous external validation on a prospective postsurgical cohort are explicitly named as prerequisites for any clinical deployment of this framework.

#### Reflexion-Specific Failure Modes

Three Reflexion-specific failure modes are acknowledged. (1) Self-confirmation bias: mitigated by the CFVL as an external factual check independent of the Reflexion loop, the N_max = 2 hard limit preventing recursive amplification, and RAG provenance tagging grounding all claims in retrieved evidence. (2) Recursive instability: The N_max limit prevents unbounded recursion; the verbatim audit trail enables post hoc identification and prompt refinement. (3) Critique-action decoupling (nominal acknowledgement without substantive revision): Targeted for empirical detection in the planned retrospective simulation; in the interim, CFVL independently checks revised traces before the Act step.

### 8.2. Latency Profile

A ReAct cycle with tool invocations and Reflexion self-evaluation introduces substantially greater latency than a rule-based policy evaluation: estimated 800–1500 ms per full cycle versus < 10 ms for the prior P(p,t) evaluation. For routine monitoring cycles (DETER observations, resource allocation review), this latency is clinically acceptable. For time-critical escalations (intraoperative haemodynamic collapse, post-surgical cardiac arrest), the fallback rule-based agent provides sub100 ms response times. A hybrid architecture—agentic LLM for routine decision-making and rule-based fallback for time-critical escalations—is the recommended production configuration.

#### Concurrent Multi-Patient Load Analysis

For 10 simultaneous postsurgical patients (estimates from published benchmarks [16,33], not validated under concurrent load): BioMistral-7B (∼14 GB VRAM FP16) needs a dual-A100 configuration (∼160 GB); Meditron-70B (∼140 GB) needs four A100s. The DETER Agent generates ∼2 reasoning requests/min at this load, against the 40–67 cycles/min a single A100 sustains (Table 7)—a 20–30× margin. Per-patient active context stays within BioMistral-7B’s 4096-token window with light compression beyond 48 h.

### 8.3. Confidence Calibration

The confidence-gated HITL mechanism depends on the LLM’s self-assessed confidence being well-calibrated. LLM confidence self-assessment is an active research problem: models tend to be overconfident in their reasoning, particularly for novel scenario types. Empirical calibration of the confidence threshold on a prospective clinical dataset—establishing what self-assessed confidence level correlates with acceptable clinical error rates—is a prerequisite for safe deployment.

The 0.75 default threshold is a pre-calibration conservative estimate, acknowledging that LLMs tend to be overconfident in self-assessment, particularly for novel scenario types. The formal calibration methodology specified in Section 7.3—including Platt scaling, isotonic regression, and clinical loss function threshold selection on the 94-patient DETER cohort—is the specified path to determining the deployment-appropriate value. The threshold is a configurable DETER DSS parameter and should be re-calibrated following any LLM update or domain expansion.

### 8.4. Illustrative Simulation Scenarios

The following four scenarios present formally specified temporal traces of agent behaviour under defined clinical conditions. These are design-level simulations demonstrating agent activation, inter-agent communication, and temporal response characteristics, based on the architectural specification and latency estimates in Table 7. They are not software-implemented traces; empirical measurement under realistic parallel patient loads is a primary objective of the planned pilot (Section 8.5) [31].

Scenario 1—Routine Postsurgical Monitoring (t = 0 to t = 480 min, post-ICU admission): The DETER Monitoring Agent performs 48 consecutive observation cycles (5 min intervals). DETER 6 h risk scores remain stable (<0.35 throughout). Agent self-assessed confidence remains > 0.75 on all cycles; Reflexion self-critiques confirm no artefacts or missing data; N_max not reached. No HITL escalations triggered. The Resource Allocation Agent approves routine postoperative blood tests and chest X-rays automatically. The Coordination Supervisor logs all agents as healthy at each 30 s heartbeat. Demonstrates nominal temporal behaviour and the alert-fatigue-reduction property: high-confidence routine actions proceed without HITL interruption.

Scenario 2—Progressive Deterioration with Multi-Agent Coordination (t = 18 h postsurgery): DETER 6 h risk score rises from 0.42 → 0.57 → 0.71 over three consecutive cycles (Δ = 0.145/cycle; ∇DETER = 0.145). Reflexion self-critique at cycle 3 identifies the rising gradient as clinically significant (troponin-elevation + low cardiac output pattern). RAG_retrieve() invoked (multi-hop: complication profile → incidence data → guideline recommendations). The DETER Monitoring Agent publishes a structured alert to the Coordination Supervisor. The Coordination Supervisor invokes conflict_resolve_P(): patient P(p,t,context) score elevated by w_5_·f_5_(0.145) = 0.0145. The Resource Allocation Agent is tasked to expedite troponin + BNP assays (Stage 1 automatic resolution). HITL triggered because DETER agent confidence = 0.68 < 0.75. Full trace from first observation to clinician notification: approximately 3200 ms.

Scenario 3—Intraoperative Haemodynamic Collapse (t = 00:47:22 operative time): MAP drops to 52 mmHg for 38 s during CPB weaning (threshold: MAP < 65 mmHg for >30 s). Rule-based fallback activates immediately (<100 ms): an immediate haemodynamic alert is published to the theatre team. Simultaneously, the Intraoperative Monitoring Agent ReAct cycle initiates. DETER_predict() runs on the current window. The LLM reasoning trace evaluates haemodynamic instability in the context of CPB weaning stage (Φ(t) surgical context vector). Differential reasoning: vasoplegic syndrome vs. ventricular dysfunction vs. hypovolaemia. Confidence = 0.61 → HITL_escalate() invoked. The LLM cycle completes, and the HITL dashboard is presented to the anaesthetist at t + 1100 ms. Safety property demonstrated: rule-based fallback ensures sub-100 ms response; LLM provides clinical context for the HITL decision 1 s later.

Scenario 4—Resource Conflict: DETER Monitoring Agent vs. Resource Allocation Agent (t = 36 h postsurgery): The DETER Monitoring Agent requests an urgent echocardiogram; patient DETER 6 h score = 0.73, ∇DETER = 0.18 (active deterioration). The Resource Allocation Agent flags the order as low-priority based on echo slot availability. The Coordination Supervisor invokes conflict_resolve_P(): f_5_(0.18) contribution elevates the patient priority score by 0.018; the resulting total priority advantage exceeds the auto-resolution threshold ε_auto = 0.05. Stage 1 automatic resolution: echo expedited. Coordination Supervisor Reflexion self-critique: confidence = 0.82, no HITL required. Chain-of-thought justification written to audit trail. Total conflict-to-resolution time: approximately 1800 ms.

Scenario 5—Postoperative New-Onset Atrial Fibrillation (t = 22 h post-CABG).

Cycle 1 (t = 22 h 03 m): DETER_predict() returns 6 h risk = 0.52; HR irregularity is the primary feature. RAG_retrieve() (postsurgical) returns AF incidence 20–40% post-CABG and the ESC 2020 requirement for a 12-lead ECG before pharmacological management; CFVL validates drug dosages. Reflexion returns CONFIDENCE: 0.64; below θ = 0.75, HITL_escalate() fires and the clinician approves an ECG at t + 1450 ms.

Cycle 2 (t = 22 h 18 m): ECG confirms AF at 118 bpm; DETER risk rises to 0.67. conflict_resolve_P() raises patient priority; the Resource Allocation Agent expedites haematology. Multi-hop RAG returns ESC 2020 and STS guidance (rate control preferred <24 h post-CABG); CFVL clears amiodarone dosing. Reflexion returns CONFIDENCE 0.79, but FHIR_observation_write() is in the mandatory HITL set, so HITL_escalate() fires regardless. Clinician approves at t + 2800 ms. Demonstrated: two-cycle confidence evolution (0.64 → 0.79), multi-hop RAG, CFVL drug-safety checking, and mandatory HITL for care-pathway changes.

### 8.5. Future Directions

Future work should: (1) implement a controlled pilot of the DETER Monitoring Agent and Resource Allocation Agent at the University of Patras Cardiothoracic Clinic, comparing LLM-mediated reasoning against clinician decisions prospectively; (2) conduct systematic adversarial testing of agent reasoning using a library of novel post-surgical cardiopulmonary scenarios not representable by the existing evidence base; (3) empirically calibrate the HITL confidence threshold on the 94-patient DETER validation cohort; (4) evaluate the latency profile under realistic parallel patient loads (target: 10 simultaneous postsurgical patients per unit); and (5) extend the agentic framework to incorporate a State-Space Model (Mamba) or Neural ODE backends in DETER_predict() for irregular-sampling and long-horizon prediction, as identified by Pylarinou and Gortzis in the companion methodological analysis [34]. (6) Retrospective Simulation Protocol: A structured simulation against the 94-patient DETER cohort [1] is planned prior to the clinical pilot. The DETER Monitoring Agent will run in replay mode, comparing escalation decisions against documented clinical events using escalation concordance (Cohen’s κ), sensitivity/specificity at the 6 h deterioration horizon, and HITL rate vs. clinician benchmark. The term “clinically deployable” throughout refers to architectural design readiness, not validated clinical readiness absent empirical evaluation.

## 9. Conclusions

We have presented the first agentic LLM framework for autonomous surgical continuum care, replacing the rule-based logic of the prior MAS Digital Twin framework [1] with six ReAct-driven, Reflexion-equipped, tool-use agents operating across the presurgical–intraoperative–postsurgical cardiopulmonary care continuum.

The architectural transformation is fundamental, not incremental. Rule-based agents execute predefined plans; agentic agents generate plans. Rule-based agents fail silently on novel scenarios; agentic agents express uncertainty and escalate. Rule-based conflict resolution applies fixed weights; agentic coordination reasons over contextual factors with chain-of-thought justification. These differences are not implementation details—they determine whether the system can function as intended in the clinical reality of postsurgical cardiopulmonary care, where novel scenario combinations arise daily and where the clinical stakes of reasoning errors are highest.

The confidence-gated HITL architecture represents a principled solution to the alert fatigue problem that has undermined clinical AI adoption: by anchoring HITL escalation to agent-expressed reasoning uncertainty rather than to fixed thresholds, the framework reduces unnecessary human interruptions for high-confidence routine actions while strengthening oversight precisely where it is most needed—when the agent itself recognises the limits of its reasoning.

The formal specifications provided—ReAct loop signatures, tool registry, extended P(p,t,context) function, safety invariants, and EU AI Act compliance mapping—establish the architectural foundation for prospective implementation and evaluation. Together with the DETER deterioration prediction algorithm [1] and the evidence-grounded RAG CDS architecture [31], this framework constitutes a complete, formally specified, and clinically deployable intelligent monitoring ecosystem for postsurgical cardiopulmonary care.

## Figures and Tables

**Table 1:** Comparative positioning of the proposed framework against prior clinical AI coordination systems.

System	Autonomy	Tool Use	Safety Enforcement	Temporal Coverage	Self-Correction
JADE/JASON BDI [6,7]	Rule-bound (L1)	Fixed APIs	Formal (plan library)	Episode	No
DETER MAS Prior [1]	Rule-bound+PPO (L1)	Fixed APIs	Formal (priority queue)	Hours–days	No
Med-PaLM 2 [15]	Single LLM (L3)	None	None	Single query	No
Hemmerling 2010 [10]	Closed-loop (L2)	PK/PD model	Rule-based	Single procedure	No
AutoGen [17]	Dynamic LLM (L4)	Dynamic	Informal	Task-specific	Minimal
MDAgents [25]	Dynamic LLM (L4)	Limited	Voting consensus	Single case	Limited
This work	Agentic LLM (L5)	Formal registry (10)	Multi-layer + CFVL	Full continuum	Reflexion+CFVL

**Table 2:** Quantitative fallback trigger thresholds by agent and clinical context.

Agent	Trigger Condition	Threshold Value	Basis
Intraoperative Monitoring	Mean arterial pressure (MAP)	<65 mmHg for >30 consecutive seconds	AAGBI/SCA intraoperative crisis criteria
Intraoperative Monitoring	Heart rate	<40 bpm or >160 bpm for >20 s	AAGBI/SCA intraoperative crisis criteria
Intraoperative Monitoring	SpO_2_	<88% for >60 s	AAGBI/SCA intraoperative crisis criteria
Intraoperative Monitoring	CPB pump flow	Drop > 30% from baseline within one observation window	Cardiothoracic surgical protocol
DETER Monitoring (ICU/ward)	DETER 6 h risk score	≥0.85 on two consecutive prediction cycles (~10–20 min)	DETER validation cohort [1]
DETER Monitoring (ICU/ward)	NEWS2 score	≥7 on single assessment	Royal College of Physicians NEWS2 [27]
DETER Monitoring (ICU/ward)	LLM inference/Reflexion limit	Timeout > 2000 ms, exception, or N_max = 2 reached without convergence	Latency budget (Section 8.2)
Coordination Supervisor	LLM conflict resolution	Fails after N_max = 2 iterations	Reflexion termination condition (Section 3.2)
Coordination Supervisor	Simultaneous HITL requests	≥3 agents requesting HITL in one coordination cycle	System load threshold
Coordination Supervisor	OPEL level	Level 4 (system black)—MCI protocol activated; agentic coordination suspended	NHS England OPEL framework
All agents (universal)	LLM inference failure	Any timeout > 2000 ms, any exception, or out-of-registry tool call attempt	Safety-by-default fallback policy

Note: All thresholds are configurable parameters in the DETER DSS, allowing site-specific calibration to local clinical protocols.

**Table 3:** Architectural comparison: rule-based MAS vs. agentic LLM framework.

Dimension	Rule-Based MAS (Prior Framework)	Agentic LLM Framework (This Work)
Agent logic	Hardcoded decision trees and PPO-trained policies per role	LLM-based autonomous agents with dynamic goal decomposition, tool invocation, and self-correction via ReAct loops
Reasoning	Deterministic if–then rules; cannot handle novel scenario combinations	Chain-of-thought + tool-use reasoning; generalises to unseen clinical presentations
Tool integration	Fixed API calls per agent role; no dynamic tool selection	Dynamic tool registry: agents select and invoke the appropriate FHIR/clinical/computational tool at runtime
Inter-agent coordination	Rule-based message passing with static priority queue P(p,t)	LLM-mediated negotiation with structured handoff protocols; P(p,t) replaced by agent-reasoned consensus under constraints
Knowledge source	Static RAG pipeline queried per fixed trigger	Agents dynamically decide when and what to retrieve; multi-hop retrieval chains for complex clinical questions
Self-correction	No; errors propagate unless HITL intervenes	Reflexion-style self-evaluation: agents critique their own reasoning before acting
Application domain	Pre-hospital to ED triage (acute, episodic)	Presurgical–intraoperative–postsurgical continuum (longitudinal, multi-phase)
Temporal horizon	Minutes to hours (ED encounter)	Days to weeks (surgical journey from consent to rehabilitation)
HITL interface	Override at decision escalation points	HITL is a mandatory gating step in every ReAct cycle before any tool execution that modifies care pathway

PPO: Proximal Policy Optimisation; HITL: Human-in-the-Loop; ReAct: Reasoning + Acting [2]; Reflexion [3]; P(p,t): multi-criteria weighted priority function.

**Table 4:** Agentic agent specification: ReAct loop, tool registry, and authorisation.

Agent	Surgical Phase	ReAct Loop Signature	Tool Registry	Authorisation and HITL Gate
Preoperative Risk Stratification Agent	Presurgical consent to scheduling	Observe: patient HIS data + FHIR records. Reason: EuroSCORE II/STS risk synthesis. Act: risk report + surgical plan endorsement or flag.	EuroSCORE_calc(), STS_risk_api(), FHIR_patient_read(), guideline_retrieve(), DETER_DSS_query(), comorbidity_screen()	Read EHR; generate risk report; HITL gate before any surgical plan modification or high-risk flag propagation
Intraoperative Monitoring Agent	Operative phase (sterile-field-aware)	Observe: real-time haemodynamic + anaesthesia streams. Reason: deterioration trajectory vs. operative stage. Act: alert escalation or physiological correction suggestion.	biosensor_stream_read(), anaesthesia_monitor_api(), CPB_event_log(), DETER_predict(), NEWS2_calc(), alert_publish()	Read monitors; generate alerts; HITL gate before any anaesthesia or perfusion protocol recommendation
DETER Monitoring Agent ★ Core	Postsurgical ICU and ward	Observe: CAREPOI telemetry 5–10 min + EMR delta. Reason: 6 h/24 h/7 d deterioration trajectory via DETER. Act: personalised risk score + CDS recommendation chain.	DETER_predict(), RAG_retrieve(), FHIR_observation_write(), NEWS2_calc(), alert_escalate(), audit_log()	Write observations; escalate alerts; HITL gate before all care pathway modifications; every act logged immutably
Resource Allocation Agent ★	All surgical phases	Observe: DT Core S(t) resource state + examination orders. Reason: evidence-based investigation appropriateness per phase. Act: approve/flag/redirect examination orders; coordinate HEART ECG.	DT_state_read(), bed_assign(), examination_order_evaluate(), LIMS_api(), RIS_api(), ECG_coordinate(), cost_track()	Read resource state; flag orders; HITL gate before bed reassignment or examination restriction affecting care
Discharge and Rehabilitation Agent	Post-discharge remote monitoring	Observe: LACE+ readiness + CAREPOI remote telemetry. Reason: trajectory-based discharge timing and follow-up intensity. Act: discharge plan + remote monitoring initiation + PROMs scheduling.	LACE_plus_calc(), FHIR_task_write(), CAREPOI_remote_init(), PROM_schedule(), follow_up_book(), EHR_writeback()	Write discharge plan; HITL gate before admission-to-discharge transition; initiate remote monitoring autonomously within authorised parameters
Coordination Supervisor Agent	System-wide (all phases)	Observe: all agent states + DT Core health + OPEL level. Reason: multi-agent conflict under priority function + surge detection. Act: re-prioritise queue; escalate to HITL for unresolvable conflicts.	agent_health_monitor(), priority_queue_manage(), conflict_resolve_P(), OPEL_read(), MCI_protocol_trigger(), HITL_escalate()	Orchestrate all agents; HITL escalation for any inter-agent conflict above epsilon threshold; MCI protocol activation

★ marks agents incorporating DETER deterioration prediction. CPB: cardiopulmonary bypass. LACE+: Length of stay, Acuity, Comorbidity, ED use index. PROM: Patient-Reported Outcome Measure. HITL gate: mandatory clinician digital-signature confirmation before care-pathway-modifying tool execution.

**Table 5:** Clinical tool registry: specification, parameters, and agent invocation.

Tool Name	Category	Description and Parameters	Invoked by
DETER_predict()	Prediction	Runs DETER Transformer inference on current 144-step physiological window. Returns: risk_score{6 h, 24 h, 72 h}, confidence_interval, and feature_importance_map.	DETER Monitoring, Intraoperative
RAG_retrieve()	Knowledge	Semantic search over the cardiopulmonary surgery literature, 97 procedure profiles, ESC/AHA/ACC guidelines, and SNOMED-CT. Params: query_text, top_k, and phase_filter. Returns: evidence_list with provenance [28].	DETER Monitoring, Preoperative Risk
FHIR_patient_read()	Data	Reads Patient, Observation, Condition, MedicationRequest FHIR R4 resources. Params: patient_id, resource_types[], and time_window. Returns: structured FHIR bundle [29].	All agents
FHIR_observation_write()	Data	Creates FHIR observation resource with HITL approval flag. Params: patient_id, observation_type, value, unit, and flag_hitl. Returns: resource_id.	DETER Monitoring
EuroSCORE_calc()	Clinical	Computes EuroSCORE II and STS risk scores from structured patient data. Returns: logistic_euroSCORE, additive_euroSCORE, STS_mortality, and STS_morbidity.	Preoperative Risk
examination_order_evaluate()	Resource	Evaluates proposed examination order against ESI-stratified evidence protocols and current resource availability from DT Core. Returns: approve|flag|redirect with justification.	Resource Allocation
conflict_resolve_P()	Coordination	Computes the extended priority function P(p,t,context) = w_1_·f_1_(acuity_p) + w_2_·f_2_(WT_p(t)) + w_3_·f_3_(CR_p(t)) + w_4_·f_4_(phase_p(t)) + w_5_·f_5_(∇DETER_p(t)) for each competing patient, with default weights w = (0.40, 0.15, 0.20, 0.15, 0.10). Returns: ranked patient list with priority scores.	Coordination Supervisor
agent_health_monitor()	Infrastructure	Polls heartbeat of all active agents. Detects timeout, exception, or deadlock. Activates backup instance on failure. Returns: agent_status_map.	Coordination Supervisor
HITL_escalate()	Safety	Packages agent reasoning chain, evidence provenance, and proposed action into structured HITL dashboard alert. Requires clinician digital signature before proceeding. Returns: approval|rejection|modified_action.	All agents (mandatory)
audit_log()	Compliance	Writes immutable timestamped record of agent_id, reasoning_chain, tools_called, evidence_retrieved, action_proposed, and HITL_outcome to DETER DSS audit trail (EU AI Act Article 12 compliant) [19,30].	All agents (mandatory)

FHIR: Fast Healthcare Interoperability Resources R4. FAISS: Facebook AI Similarity Search. LIMS: Laboratory Information Management System. RIS: Radiology Information System. HITL: Human-in-the-Loop. EU AI Act Article 12: obligation to maintain logs for high-risk AI systems.

**Table 6:** Conflict resolution: prior MAS framework vs. agentic extension.

Component	Prior MAS Framework	Agentic Framework (This Work)	Rationale
Priority function	P(p,t) = w1*f1(ESI) + w2*f2(WT) + w3*f3(CR)	Extended: P(p,t,context) += w4*f4(surgical_phase) + w5*f5(DETER_trajectory_gradient)	Surgical phase modulates urgency beyond acute ESI; DETER gradient captures rate of deterioration, not just current level
Conflict resolution	Rule-based Tier 2 Priority Queue Manager	Agent-mediated: Coordination Supervisor LLM reasons over competing requests, invokes conflict_resolve_P(), and proposes resolution with chain-of-thought justification	LLM reasoning handles novel conflict types not encodable in fixed weight vectors
HITL escalation trigger	Fixed threshold: |P(p) − P(p’)| < epsilon = 0.01	Dynamic: Agent requests HITL when self-assessed confidence < 0.75 OR when two competing resources have equal priority OR when novel scenario type detected	Confidence-gated HITL reduces alert fatigue while preserving safety for genuinely ambiguous situations
Deadlock handling	Supervisor activates backup agent instance	Reflexion loop: Coordination Supervisor critiques own conflict resolution, attempts re-reasoning with broader context before escalating to HITL	Self-correction before escalation reduces unnecessary human interruptions
MCI surge mode	OPEL term added to P: P_MCI += w4*f4(OPEL)	Autonomous protocol switch: MCI Coordinator sub-agent instantiated dynamically; full surgical care pathway suspended in favour of damage-control prioritisation	Agentic architecture allows runtime agent instantiation for novel scenarios

**Table 7:** Temporal characteristics of individual agents.

Agent	Observation Polling	ReAct Cycle (est.)	Reflexion Overhead	Latency Regime
Preoperative Risk Stratification	On-demand at surgical consent	1200–2000 ms (EuroSCORE + STS + RAG multi-hop)	800–1200 ms	Non-time-critical (hours to days before surgery)
Intraoperative Monitoring	60 s window; continuous CPB event monitoring	800–1500 ms (routine); <100 ms (rule-based fallback, time-critical events)	600–900 ms; N_max = 2	Time-critical; dual-path hybrid
DETER Monitoring Core	5–10 min CAREPOI telemetry cycle	900–1500 ms	700–1100 ms	Routine ICU/ward; <100 ms fallback for DETER score ≥ 0.85
Resource Allocation	Event-driven (examination order receipt)	700–1200 ms per order	500–800 ms	Non-time-critical
Discharge and Rehabilitation	Daily LACE+ + PROM cycle	1000–1800 ms	700–1000 ms	Non-time-critical; mandatory HITL adds clinician response time
Coordination Supervisor	Continuous agent heartbeat (30 s intervals)	1000–1500 ms for conflict resolution	800–1200 ms	Continuous; rule-based fallback on LLM failure < 50 ms

All latency estimates are derived from published BioMistral-7B and Meditron-70B inference benchmarks on GPU-accelerated hardware, plus FHIR R4 and DETER DSS API round-trip estimates [16,33]. Empirical measurement under realistic parallel patient loads is a primary objective of the planned pilot (Section 8.5).

## Data Availability

No datasets were generated in this study. The companion DETER dataset (94 patients, University Hospital of Patras) is available from the corresponding author on reasonable request.
